# Molecular Characterization and Biocompatibility of Exopolysaccharide Produced by Moderately Halophilic Bacterium *Virgibacillus dokdonensis* from the Saltern of Kumta Coast

**DOI:** 10.3390/polym14193986

**Published:** 2022-09-23

**Authors:** Monic Andrew, Gurunathan Jayaraman

**Affiliations:** School of Biosciences and Technology, Vellore Institute of Technology, Vellore 632014, Tamil Nadu, India

**Keywords:** halophiles, marine bacteria, exopolysaccharides, fermentation, structural characterization, biomaterial, anticoagulant activity, hemocompatibility, cytocompatibility

## Abstract

The use of natural polysaccharides as biomaterials is gaining importance in tissue engineering due to their inherent biocompatibility. In this direction, the present study aims to explore the structure and biocompatibility of the EPS produced by *Virgibacillus dokdonensis* VITP14. This marine bacterium produces 17.3 g/L of EPS at 96 h of fermentation. The EPS was purified using ion exchange and gel permeation chromatographic methods. The porous web-like structure and elemental composition (C, O, Na, Mg, P, S) of the EPS were inferred from SEM and EDX analysis. AFM analysis revealed spike-like lumps with a surface roughness of 84.85 nm. The zeta potential value of −10 mV indicates the anionic nature of the EPS. Initial molecular characterization showed that the EPS is a heteropolysaccharide composed of glucose (25.8%), ribose (18.6%), fructose (31.5%), and xylose (24%), which are the monosaccharide units in the HPLC analysis. The FTIR spectrum indicates the presence of functional groups/bonds typical of EPSs (O-H, C-H, C-O-H, C-O, S=O, and P=O). The polymer has an average molecular weight of 555 kDa. Further, NMR analysis revealed the monomer composition, the existence of two α- and six β-glycosidic linkages, and the branched repeating unit as → 1)[α-D-Xyl*p*-(1 → 2)-α-D-Glc*p*-(1 → 6)-β-D-Glc*p*-(1 → 5)]-β-D-Fru*p*-(2 → 2)[β-D-Xyl*p*-(1 → 4)]-β-D-Xyl*p*-(1 → 6)-β-D-Fru*f*-(2 → 4)-β-D-Rib*p*-(1 →. The EPS is thermally stable till 251.4 °C. X-ray diffraction analysis confirmed the semicrystalline (54.2%) nature of the EPS. Further, the EPS exhibits significant water solubility (76.5%), water-holding capacity (266.8%), emulsifying index (66.8%), hemocompatibility (erythrocyte protection > 87%), and cytocompatibility (cell viability > 80% on RAW264.7 and keratinocyte HaCaT cells) at higher concentrations and prolongs coagulation time in APTT and PT tests. Our research unveils the significant biocompatibility of VITP14 EPS for synthesizing a variety of biomaterials.

## 1. Introduction

The demand for biomaterials and their predominant usage in several biomedical and clinical applications has increased significantly across the world over the last few years. Currently, natural polysaccharides have been the focus of research in pharmaceutical and biomedical fields [1]. Contemporary research on marine resources has unearthed many excellent bioactive properties of polysaccharides for developing valuable biomaterials. In fact, marine biomaterials have fascinated a wide range of industries, from engineering to pharmaceuticals and cosmetics, thus far [2]. These natural polysaccharides are of major interest in tissue engineering and biomaterial synthesis as they are widely available, low or non-toxic, biodegradable, and more ecofriendly than synthetic polymers [3]. Due to ubiquitous biocompatibility and good mechanical properties, natural polysaccharide-based biomaterials are utilized in the development of tissue scaffolds, implantation, artificial grafts, wound fabrication, drug delivery, and bone filler materials. Significantly, they are easily accepted by the cells and tissues of the human body [4]. In search of efficient biocompatible natural polysaccharides, bacterial exopolysaccharides (EPSs) are being investigated in detail due to their sustainability, fast production, and ecofriendly properties. EPSs are high-molecular-weight, organic biomacromolecules secreted in the natural environment by the host organism and are involved in the defense, symbiosis, phagocytosis, signaling process, cell development by nutrient entrapment, and prevention of desiccation. Generally, EPSs are composed of several functional groups, diverse monosaccharide composition, uronic acids, and also a few non-carbohydrate constituents [5,6]. Owing to their therapeutic potential, bacterial EPSs are being exploited in various applications as ingredients in nutraceuticals, pharmaceuticals, and cosmetics [7]. Health-promoting effects of bacterial EPSs, such as being an antioxidant [8], antiviral, antibacterial, antitumor, anticoagulant, anti-inflammatory, immune-regulating, and antibiofilm, as well as being used for vaccine development and drug delivery [9], are also being investigated. Bacterial EPSs also have great potential for use in effective skin and bone regeneration as well as in the synthesis of various scaffolds [5,10]. However, hemocompatibility and cytocompatibility are crucial indicators which limit the clinical acceptability of biomaterials. The adverse interaction of biomaterials with blood components or cells can damage the blood cells and tissues. Hence, these properties should be analyzed while developing a suitable biomaterial [10,11]. Therefore, the necessity of superior biocompatible molecules with the desired structure and physicochemical properties is absolutely essential for successful application in the biomedical industry, especially for tissue engineering. In this regard, the attributes of EPSs with low or non-cytotoxicity, biocompatibility, high thermal stability, an accepted gelation property, good water retention capacity, and the possibility for facile structural modifications, favor them for applications in biomedical and tissue engineering.

Despite the promising application of EPSs in diverse fields, information pertaining to the diversity of halophilic microorganisms explored for producing these biopolymers is inadequate. Even though the production of EPSs is prevalent among microorganisms, they have been mostly derived from non-halophilic bacteria. Halophilic microorganisms from saline and hypersaline environments often harbor both moderately and extremely halophilic bacteria of biotechnological interest. However, they are far from being explored and exploited for the production of EPSs [12]. Halophilic bacteria are salt-loving extremophilic microorganisms and are categorized as slight halophiles, moderate halophiles, and extreme halophiles, depending on the salt concentrations (3–25% (*w*/*v*)) required for optimal growth [13]. These inhabitants of saline or hypersaline environments such as salt pans, salt mines, and marine ecosystems have attracted microbial biotechnologists globally due to the high intracellular concentration of inorganic ions and compatible solutes that are vital for their adaptation. They have the potential to grow in unsterile and hypersaline conditions that can facilitate a continuous fermentation process [14], which is cost-effective. To the best of our knowledge, the literature on the structure and functional characteristics of the halophilic bacterial EPSs is still limited. Therefore, to expand and explore the calibers of untapped bacterial EPSs and their application prospects, it is necessary to investigate the properties of newly discovered EPSs produced by the halophilic strains. Indeed, the coastline and marine ecosystem of the Arabian Sea have been considered potential sites with substantial ecological significance and high species richness. Since these regions have high saline and moderate thermal conditions, the authors have hypothesized that the halophilic bacterial strains present in these habitats can have significant biotechnological potential. With this background, the research work involves extraction and molecular characterization of EPSs from a halophilic bacterium, isolated from the Kumta coast (14.26° N and 74.4° E), and evaluation of biocompatibility for plausible biotechnological applications.

## 2. Materials and Methods

### 2.1. Bacterial Strain and Screening of EPS-Producing Capacity

The bacterial strain used in this study was previously isolated from water samples [15] collected from a saltern region in Kumta, the Arabian Sea Coast of India (5% *w*/*v* to 10% *w*/*v* NaCl) and the strain was maintained in our laboratory. *Virgibacillus dokdonensis* VITP14 has a 99% sequence similarity (16S rRNA) with *Virgibacillus dokdonensis* Ro79 [16]. In the current study, this bacterial isolate was evaluated for EPS production by inoculating it in Zobell marine agar (peptone, yeast extract, magnesium chloride, sodium sulfate, calcium chloride, agar, fortified with 2% *w*/*v* NaCl, pH 7.5). After 24–48 h incubation at 37 °C, the plate was checked for the presence of mucoid-aspect colonies. These mucoid colonies were picked and grown in 100 mL of Zobell marine broth maintained at 37 °C for 48 h with the agitation of 130 rpm to test the ability to produce EPS [17]. Growth media and reagents used in the study were of analytical grade (HiMedia Laboratories, Mumbai, India).

### 2.2. Growth of Bacterial Strain and EPS Production

The bacterial culture for EPS production was grown and maintained as batch cultures. Actively growing cultures were inoculated (2% *v*/*v*) separately in 100 mL (250 mL flask) of Zobell marine broth (peptone, yeast extract, magnesium chloride, sodium sulfate, calcium chloride, 2% *w*/*v* NaCl, pH 7.5) and incubated at 37 °C on a rotary shaker (130 rpm). The strain was cultured for 96 h and growth conditions (temperature and agitation) were kept unchanged. Further, the EPS production was investigated using different percentages (1%, 1.5%, 2%) of various carbon sources (glucose, fructose, lactose, maltose, sucrose) and sodium chloride (3–7%) fortified in Zobell marine broth. Subsequently, the cultures were centrifuged (10,000 rpm for 15 min at 4 °C) to collect the EPS from the supernatant, and then the EPS was quantified as per the dry weight. Next, bacterial pellets were collected and dried at 60 °C for 24 h. Then, the cell dry weight of the biomass was measured. Aliquots were drawn at different time intervals from 0 to 96 h and the bacterial growth was inferred from the changes in optical density at 600 nm, using a UV–visible spectrophotometer (Systronics AU2701, Ahmedabad, India).

### 2.3. Extraction and Purification of EPS

After 96 h of growth, the culture supernatant was centrifuged (15,000× *g*, 20 min, 4 °C). The cell-free supernatant was treated with three volumes of chilled ethanol and kept overnight at 4 °C. The precipitate was collected by centrifugation (10,000× *g*, 20 min, 4 °C) and was deproteinized by adding the Sevag reagent (1:4 *n*-butanol/chloroform, *v*/*v*). The precipitated proteins were removed by centrifugation (6000 rpm for 10 min). The EPS was precipitated by adding 2 volumes of ethanol into the deproteinized solution, which was kept for 14 h at 4 °C. Then, the mixture was centrifuged at 6000 rpm for 10 min. After centrifugation, the precipitate was re-dissolved in distilled water and the excess salts were removed by dialysis (14 kDa, HiMedia, Mumbai, India) for 24 h (at 4 °C) against distilled water. After dialysis, the retentate was lyophilized to obtain the EPS [18].

The EPS was purified by ion exchange chromatography [19] using the DEAE Cellulose-52 (HiMedia Laboratories, Mumbai, India). The EPS was eluted with a linear gradient of 0.1–1.1 mol/L NaCl with a flow rate of 1 mL/min. Fractions containing EPS were dialyzed, concentrated, and refractionated using the Sephacryl S-300 HR column (column dimension 1.8 cm × 50 cm, flow rate 1 mL/min, GE Healthcare Life Science, Uppsala, Sweden). Fractions containing a significant quantity of carbohydrates were pooled together and lyophilized. The total carbohydrate was determined by the phenol sulfuric acid method using glucose as the standard [20]. Protein content was determined with bovine serum albumin as the standard [21].

#### Characterization of EPS

The physical and chemical characterizations of the EPS were performed using various analytical techniques as per the standard protocols given in the literature [22,23,24,25,26,27,28].

### 2.4. Scanning Electron Microscopy, Energy Dispersive X-ray, and Atomic Force Microscopy

Scanning electron microscopy (SEM) was used to determine the morphology of the EPS. Dry EPS (5 mg) samples were glued on specimen stubs by coating with a thin layer of gold-palladium under high vacuum conditions and analyzed using a scanning electron microscope (Carl Zeiss, Oberkochen, Germany). The elemental composition of the EPS was determined by using energy-dispersive X-ray spectroscopy EDX (Oxford, UK).

For AFM studies, the EPS (5 mg) was dissolved in Milli-Q water and stirred to obtain a uniformly dispersed EPS solution. Subsequently, 20 μL of EPS solution was dispersed on a glass slide and dried at room temperature. The AFM images were obtained by a scanning probe microscope (NaioAFM, Nanosurf AG, Liestal, Switzerland). The cantilever oscillation frequency was 158 kHz and the driving amplitude was 0.430 V. The Gwyddion 2.61 software (Czech Metrology Institute, Jihlava, Czech Republic) was used to analyze the images.

### 2.5. Zeta Potential

The zeta potential of the EPS (1 mg/mL ultrapure water) was determined at 25 °C using a NanoPlus Zeta Potential (Micromeritics Instrument Corporation, Norcross, GA, USA) and a particle size analyzer (HORIBA SZ-100; Horiba, Kyoto, Japan).

### 2.6. High-Performance Liquid Chromatography

The type of monosaccharide in VIT14 EPS was determined using high-performance liquid chromatography (HPLC). Briefly, the purified EPS (20 mg/mL) was hydrolyzed using 4.0 M trifluoroacetic acid (TFA) (4 h at 100 °C). After hydrolysis, the TFA was eliminated using a rotary vacuum evaporator. Subsequently, 1 mL of 4.0 M NaOH was added to sample tubes. The resulting EPS was taken for further derivatization. Then, 40 μL of 0.5 M PMP (1-phenyl-3-methyl-5-pyrazolone) was added, and the tubes were placed in a water bath maintained at 70 °C for 90 min. After subsequent incubation at 4 °C for 10 min, 60 μL of 0.3 N HCl was added to stop the reaction. Chloroform (1 mL) was added to extract the free PMP (three times) [29]. The sample was filtered through a 0.22 μm membrane and was injected into the Agilent 1200 HPLC system (Palo Alto, Santa Clara, CA, USA) with an Eclipse Plus C_18_ column (4.6 mm × 250 mm, 5 μm, Agilent). The chromatographic separation of PMP derivatives was carried out using acetonitrile in distilled water (10%) and TFA (0.05%) as a mobile phase with a flow rate of 0.8 mL/min. The column was maintained at 40 °C and the eluents were detected by measuring the absorption at 245 nm. Commercial arabinose, glucose, galactose, mannose, fructose, xylose, and ribose were also subjected to identical treatment as that of the EPS. Retention time and area under the peak were used to identify and quantitate the monosaccharide units in the EPS.

### 2.7. Fourier Transform-Infrared Spectroscopy (FT-IR)

The EPS (1 mg) was mixed with 99 mg of dry KBr powder and was pelleted by pressing the mixture. Subsequently, the FT-IR spectrum was recorded on the Thermo Nicolet iS50 with an inbuilt ATR spectrometer (Shimadzu, Kyoto, Japan) over the range 4000 cm^−1^ to 400 cm^−1^.

### 2.8. Molecular Weight Determination

The molecular weight of the EPS was determined by size-exclusion chromatography (GPC, Waters GPC system with a Refractive Index Detector (RID) of 2000 infinity). The column, PL aquagel -OH 40 (300 mm × 7.5 mm), was attached to a guard column, the PL gel OH guard, 8 μm (50 mm × 7.5 mm). The EPS (10 mg/mL) was dissolved in 1 mL of 0.1 M NaNO_3_ under room temperature. A filtered sample (100 μL) was injected into the column and eluted with NaNO_3_ at a flow rate of 1 mL/min. The dextrans of diverse molecular weights were used as standards to calibrate the column. The number-average molecular weight (*M*n) and weight-average molecular weight (*M*w) were inferred, and the polydispersity index was calculated (*I* = *M*w/*M*n).

### 2.9. Nuclear Magnetic Resonance Spectroscopy

^1^H NMR, ^13^C NMR, ^1^H-^1^H COSY, ^1^H-^1^H TOCSY, ^1^H-^13^C HSQC, and ^1^H-^13^C HMBC spectra of the EPS were recorded in D_2_O using a Bruker AVANCE spectrometer operating at 400 MHz (Billerica, MA, USA). Approximately 30 mg of sample was dissolved in 0.5 mL of D_2_O (99.9%) in an NMR tube (5 mm diameter). Chemical shifts were expressed in parts per million (ppm) with reference to tetramethylsilane. MestReNova 14.3.0 NMR software (Mestrelab Research, Santiago, Spain) was used for processing and analysis of the data.

### 2.10. Thermogravimetric Analysis

The thermogravimetric analysis (TGA)/differential thermal analysis (DTA) of the EPS was carried out on a SDT Q600, Bifilar Wound furnace type V20.9 Build 20 TG-DTA (TA Instruments, New Castle, DE, USA). The EPS (1.7 mg) was placed in a platinum crucible and heated at a linear rate of 20 °C/min over a temperature range of 30 °C to 800 °C under nitrogen, and the corresponding weight loss was determined as per the standard procedures.

### 2.11. X-ray Diffraction Analysis

To determine the physical characteristics of the EPS, X-ray diffraction (XRD) was recorded using a powder diffractometer (Bruker, D8 Advance) [30]. Scanning was carried out at ranges of 2θ angles (20–70°). The crystallinity index (*CI*) was determined by the ratio of the area under the crystalline peaks to the total area of the scattered diffractogram.

$$CI\;\%=\frac{\sum A\;Crystal}{\sum A\;Crystal+\sum A\;Amorphous}\times 100$$

### 2.12. Water Solubility Value, Water-Holding Capacity, and Emulsifying Activity

#### 2.12.1. Water Solubility Value

The solubility of the EPS in water was determined following the method described by Guangbin Ye et al. [31]. Briefly, 500 mg of the EPS was dissolved in 10 mL of distilled water and heated for 2 h at 80 °C. The sample was then cooled to room temperature and centrifuged at 3000× *g* for 10 min. The EPS was precipitated from the supernatant using cold ethanol as mentioned before. The powder, containing the EPS, was vacuum-dried at 50 °C and weighed. The percentage of solubility is calculated as:

Solubility (%) = [total sample weight in supernatant/dry weight] × 100

#### 2.12.2. Water-Holding Capacity

The water-holding capacity (WHC) of the EPS was determined using a slightly modified method by Gan et al. [32]. Briefly, 3 mL of ultrapure water was gradually added to 500 mg of freeze-dried EPS in a pre-weighed centrifuge tube and vortexed for 2 min to obtain a uniform dispersion. The solution was then centrifuged at 10,000× *g* for 10 min. Subsequently, the supernatant was decanted and the tube was weighed. The WHC was calculated as follows:

Water-holding capacity (%) = [Water bound weight of EPS/Initial sample weight] × 100

#### 2.12.3. Emulsifying Activity

The emulsifying activity of the EPS was evaluated as per the procedure described by Nadia et al. [33]. Briefly, 3 mL of olive oil was added to 2 mL of the EPS (1%, *w*/*v* water) or Tween 80 (control) in closed glass test tubes, and the contents were vortexed to homogeneity and incubated at room temperature for two different periods (24 and 48 h). Subsequently, the emulsion index (E_24_ and E_48_) was determined according to the formula given below. The %EA was calculated by dividing the measured height of the emulsion layer (in cm) by the total height of the mixture (in cm).

Emulsifying activity (%) = [h_e_/h_t_] × 100

where h_e_—the height of the emulsion layer; h_t_—the total height of the mixture.

All samples were stored at room temperature.

### 2.13. Hemocompatibility and Erythrocyte Membrane stabilization Activity

#### 2.13.1. Preparation of Suspension (10% *v*/*v*) of Human Red Blood Cell

Blood (10 mL) was collected from a healthy volunteer with their personal consent and transferred to heparinized centrifuge tubes. Tubes were then centrifuged at 3000 rpm at room temperature for 12 min. The supernatant (plasma and leucocytes) was decanted and the packed red blood cells were washed with saline (0.85% *w*/*v* NaCl). The process of washing and centrifugation was repeated three times until the supernatant was clear. Erythrocyte suspension (10% *v*/*v*) was used for further studies.

#### 2.13.2. Hemolytic Activity Assay

The hemolytic activity of the EPS was determined from the leakage of hemoglobin from human red blood cells [34]. Briefly, the EPS (50, 100, and 500 µg/mL) was added to the erythrocyte suspension (0.5 mL) and then incubated for 30 min at 37 °C. After the incubation, the mixture was centrifuged for 10 min at 2000 rpm and the absorbance of the supernatant was measured at 560 nm using a Systronics AU2701 double-beam spectrophotometer (Ahmedabad, India). Triton-X100 0.1% was used as positive control for 100% hemolysis. The percentage of hemolysis was determined using the equation:

Hemolysis (%) = (OD of test/OD of control) × 100

#### 2.13.3. Hypotonic Solution-Induced Hemolysis

The membrane-stabilizing activity of the EPS was determined as described by Abbou et al. [35]. The assay mixture consisted of washed stock 10% (*v*/*v*) erythrocyte (RBC) suspension (0.5 mL), 2 mL of hypotonic solution (0.3% *w*/*v*) NaCl, 1 mL of sodium PBS (pH 7.4), and the EPS (50, 100, and 500 µg/mL). The mixture was incubated for 30 min at 37 °C, centrifuged for 10 min at 2000 rpm, and the absorbance of the supernatant was measured at 560 nm using the Systronics AU2701 double-beam spectrophotometer (Ahmedabad, India). Diclofenac sodium (50–500 µg) was used as the standard. The percentage of hemolysis was determined using the equation:

Hemolysis (%) = (OD of test/OD of control) × 100

Then, the percentage of human erythrocyte membrane stabilization or protection was calculated by using the following equation:

Protection (%) = 100 − (OD of test)/(OD of control) × 100

where OD is the optical density; RBC suspension in hypotonic solution without drug or EPS (control); RBC suspension in hypotonic solution with drug or EPS (test).

#### 2.13.4. Heat-Induced Hemolysis

A heat-induced hemolysis assay was performed as per the method described by Vidhyalakshmi et al. [36]. The assay mixture consisted of 0.5 mL of 10% (*v*/*v*) erythrocyte (RBC) suspension with or without the EPS (50–500 µg/mL). Diclofenac sodium (50, 100, and 500 µg) was used as the standard. The reaction mixture was incubated in a water bath maintained at 60 °C for 30 min. After incubation, the tubes were cooled under running tap water, then centrifuged at 2000 rpm for 10 min, and the absorbance of the supernatant was recorded at 560 nm using the Systronics AU2701 double-beam spectrophotometer (Ahmedabad, India). The percentage of heat-induced hemolysis was calculated using the following equation:

Hemolysis (%) = (OD of test/OD of control) × 100

Then, the percentage of human erythrocyte membrane stabilization or protection was calculated by using the following equation:

Protection (%) = 100 − (OD of test)/(OD of control) × 100

where OD is the optical density; heat-induced RBC suspension without drug or EPS (control); heat-induced RBC suspension with drug or EPS (test).

### 2.14. Anticoagulant Activity

The anticoagulant activity was determined by measuring the activated partial thromboplastin time (APTT) and prothrombin time (PT) using heparin as a positive control, as per the method described by Song et al. (2019) with slight modifications [37]. In brief, different concentrations of the EPS were prepared (50, 100, and 500 μg/mL) by dissolving the required amount of the EPS in 0.9% NaCl. Human blood was collected from healthy donors in a tube containing 2.5% sodium citrate (9:1 *v*/*v*) and centrifuged at 3000 rpm for 20 min to separate the plasma from blood cells at 4 °C. For the APTT assay, the citrated plasma (100 μL) was mixed with 100 μL of EPS and incubated at 37 °C for 1.0 min. The APTT reagent (100 μL) was added to the mixture and incubated for 3.0 min at 37 °C. The clotting time was determined after adding 50 μL of 0.025 mM CaCl_2_ solution. For the PT assay, 100 μL of citrated plasma was mixed with 100 μL of EPS and incubated at 37 °C for 1 min, and then, 200 μL of the pre-warmed PT assay reagent was added and the clotting time was recorded using a coagulometer. Heparin sodium was purchased from HiMedia Laboratories, Mumbai, India. The assays were performed in triplicates and the results represent the mean values ± standard deviations.

### 2.15. In Vitro Cytocompatibility

RAW264.7 murine macrophages were obtained from NCCS, Pune, and HaCaT cells (provided by Christian Medical College, Vellore, India). All cells were cultured in a DMEM medium supplemented with 10% (*v*/*v*) FBS and 1% (*v*/*v*) antibiotic and antimycotic solution at 37 °C in a humidified atmosphere containing 5% (*v*/*v*) CO_2_. The EPS was dissolved in the DMEM medium with FBS at different concentrations (50, 100, 500, and 1000 μg/mL) and filter-sterilized before use. DMEM (Dulbecco’s Modified Eagle Medium) was purchased from HiMedia Labs; FBS was commercially procured from Sigma Chemicals (Balcatta, WA, Australia).

#### Cell Viability Assay

The cytocompatibility of the EPS was evaluated on RAW264.7 murine macrophages and HaCaT human keratinocytes using the MTT method [38]. In brief, 5 × 10^3^ cells/mL were seeded into a 96-well microplate and incubated at 37 °C for 24 h. Cells were then treated with different concentrations (50, 100, 500, and 1000 μg/mL) of the EPS for 24 h. Cells treated with only DMEM medium served as the control. After treatment, cells were loaded with 20 μL of MTT solution (5 mg/mL), followed by incubation for 4 h at 37 °C. Dimethyl sulfoxide (DMSO; 150 μL) was used to dissolve the purple formazan crystals. The optical density (OD) was recorded at 570 nm. The percentage of cell viability was calculated as follows:

Cell viability % = OD_sample_/OD_control_ × 100

### 2.16. Statistical Analysis

Data are expressed as mean ± SD of triplicate experiments. The statistical analysis for anticoagulant activity and cell viability was carried out by a one-way ANOVA, followed by Tukey’s test, whereas a two-way ANOVA was carried out for the membrane stabilization assay, which was followed by Dunnett’s test using GraphPad Prism version 8.0 (GraphPad Software, San Diego, CA, USA). The data are considered statistically significant if *p* < 0.05.

## 3. Results and Discussion

### 3.1. Bacterial Strain and EPS-Producing Capacity

Based on our previous reports [15], the strain used in the present study is halotolerant in nature. Moreover, this isolate (NCBI GenBank ID HQ829428) is Gram-positive and belongs to the Bacilli subclass of *Firmicutes.* Remarkably, VITP14 was studied previously for salt tolerance (0%, 5%, and 10% NaCl) potential and cell growth in both minimal media (M9) and complex Luria Broth (LB) medium. The optimal salt concentration of this strain is 5% *w*/*v* NaCl (≈0.85 M). Based on previous NMR metabolic characterization studies, this isolate produces compatible solutes such as amino acids, sugars, methyl amines, and their derivatives, which assisted with maintaining their osmotic balance in the salt stress [39,40]. In the present study, the slimy appearance and presence of mucoid bacterial colonies on agar plates were used for identifying the EPS-producing ability. Morphologically, the colonies of *Virgibacillus dokdonensis* VITP14 are creamy, smooth, and translucent, having a raised circular form as visualized on the Zobell marine agar plate (Supplementary Figure S1a,b). Further macroscopic observation indicated ropy strands in the liquid broth, which is a characteristic feature of EPS-producing organisms. These preliminary results reveal that the strain used in the present study has the potential to produce an EPS.

### 3.2. Bacterial Growth and EPS Production

**Figure 1:**
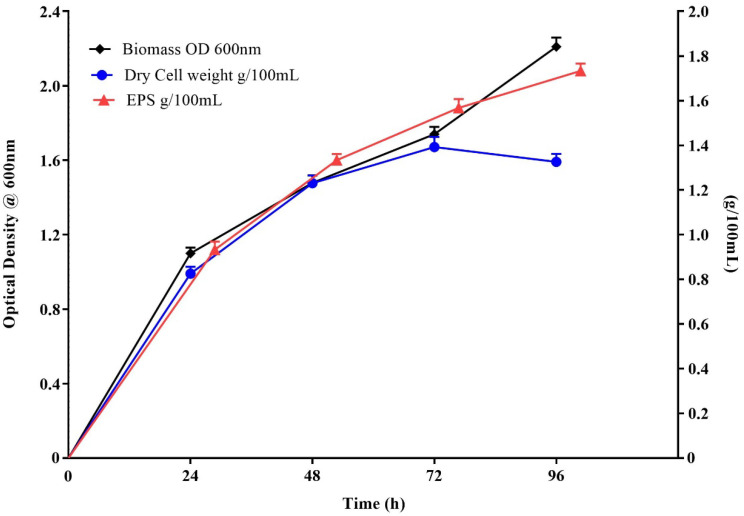
Biomass optical density (OD), the cellular dry weight (CDW), and exopolysaccharide (EPS) production as a function of time. *Virgibacillus dokdonensis* VITP14. Values are means and error bars indicate standard deviations (*n* = 3).

Both physical and chemical growth conditions are indispensable for stimulating cell growth and EPS production. Initially, microbial growth and EPS production were investigated in Zobell marine broth. Changes in cell density, dry cell weight, and EPS production were recorded periodically. EPS biosynthesis in Zobell marine broth occurs during the exponential growth period (Figure 1) and continues even during the stationary phase (even after 48 h). The amount of EPS produced by this halophilic strain reaches 13 g/L after 48 h of incubation. The maximum production of EPS (17.3 g/L) is at 96 h of growth in Zobell marine broth (Table 1). Furthermore, the biomass at 96 h reaches 13 g/L (Figure 1). Subsequently, the EPS production in the presence of various carbon sources and salt conditions was studied at a fixed growth time of 96 h. The strain produces 23.2 g/L of EPS in the presence of 2% glucose and 2% NaCl. Similarly, EPS production is 22–23 g/L in the presence of other carbon sources (2% of fructose/lactose/maltose/sucrose) (Supplementary Figure S2a). However, EPS production is only 17.3 g/L in the absence of any of these specific carbon sources, indicating that different carbon sources promote EPS synthesis by this moderately halophilic bacterium. Additionally, EPS production increases upon increasing the NaCl concentration (21 g/L at 5% NaCl and 1% glucose in Zobell marine broth). Further, an increase in NaCl results in a reduction in EPS production (Supplementary Figure S2b), indicating the presence of 5% NaCl for the maximum production of EPSs by this moderately halophilic strain. A high salt concentration in the medium can decrease the metabolic activity of moderately halophilic bacteria due to inadequate adaptation [41]. *Micrococcus roseus* produces 8 g/L after 96 h of fermentation [42] and BM39 *Pantoea* sp., a slightly halophilic bacterium obtained from the Tyrrhenian Sea, produces 21 g/L of EPSs in 24 h on the glucose medium during the exponential growth phase [43].

In the present study, the EPS production is associated with both exponential as well as stationary phases. The results suggest a strong correlation between bacterial growth and EPS production. Therefore, the strain VITP14 produces 17.3 g/L of EPS without additional carbon sources in the Zobell marine broth and 23.2 g/L of EPS upon supplementing it with 2% glucose in the Zobell marine broth. The amount of the EPS produced by the strain is comparable to those reported for other bacterial strains (Supplementary Table S2), and therefore, can be considered for industrial exploitation. However, further statistical optimization studies are in progress to determine the optimum conditions for the maximum production of the EPS.

### 3.3. Purification

The EPS obtained through ethanol precipitation was purified using ion exchange and gel filtration chromatography, and the chromatograms are given in Supplementary Figure S3. The percentage of carbohydrate and protein content present in the EPS is given in Supplementary Table S1.

### 3.4. Microstructure and Elemental Analysis of EPS

**Figure 2:**
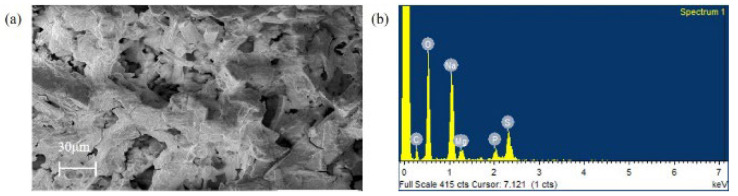
(**a**) scanning electron micrograph of exopolysaccharide (EPS) with a voltage of 10 kV under image magnification of 500×. (**b**) SEM–EDX spectrum of exopolysaccharide (EPS).

As seen from the scanning electron micrograph (Figure 2a), the EPSs produced by the bacterial strain appear as irregular lumps of different sizes with a branched morphology. SEM micrographs of EPSs reveal porous structural units and these structural arrangements contribute to the physical properties of EPSs.

An EDX analysis (Figure 2b) of the EPS reveals the presence of 21.9% carbon, 44.8% oxygen, 9.9% sulfur, 18% sodium, 3% phosphate, and 2.2% magnesium. The presence of sulfur (S) in the EPS is suggested to be the characteristic feature of marine bacteria that could be in the form of sulfate groups, which can modulate the required biological activity [44]. Inherently, marine bacterial EPSs are known to adsorb different ions (Na^+^, Mg^2+^, Ca^2+^, K^+^, Sr^2+^, Si^4+^) from seawater [45]. The elemental composition also affects the structural and functional properties of EPSs [46].

The AFM analysis is used widely to study the morphological topography of polymers. The 3D AFM image shows the formation of chains having round lumps with almost a similar size and irregular shapes when measured at 800 nm (Supplementary Figure S4). The molecules are tightly packed and their reticulated shapes describe the rough surface of the EPS. The average roughness of 84.85 nm suggests that the EPS has good roughness on its surface. It has been suggested that the rough surface of polymers promotes a stronger attachment or binding to cells and biomolecules than smooth surfaces [47]. The AFM analysis of *Bacillus licheniformis* shows a roughness average of about 191 nm [48], whereas the EPS from *Oceanobacillus iheyensis* BK6 has a roughness average of 60.61 nm [49]. It has to be noted that the surface morphology and roughness depend on the molecular structure of the EPS.

### 3.5. Zeta Potential

Zeta potential is also an important parameter to determine the ionic nature and characteristics of polysaccharides. The EPS isolated in the present study has a negatively charged zeta potential value of −10.7 mV, which indicates the acidic nature of this EPS (Supplementary Figure S5). EPSs reported to date have a wide range of negative zeta potential values. For instance, the EPS obtained from the bacteria *Enterococcus* sp. F2 has a zeta potential value of −3.44 mV [50], whereas the EPS from *Paenibacillus tarimensis* REG 0201M has a zeta potential value of −35.2 mV, which indicates their different anionic characteristics. Additionally, it has been suggested that the anionic functional groups (due to sulfates and phosphates) attached to the sugar ring impart these negative charges in EPSs [51].

### 3.6. HPLC Analysis

The HPLC chromatogram of the hydrolyzed EPS (Supplementary Figure S6a) indicates that the VIT14 EPS consists of glucose, ribose, fructose, and xylose as major sugars, whereas sugars such as arabinose, mannose, and galactose are absent. The monosaccharide units were identified based on their retention times and their relative amount was determined based on the area under the peak (Table 2). The HPLC profile of the standard monosaccharides used in the study is given in Supplementary Figure S6b. In this study, monosaccharides were selected based on the reported occurrence in EPSs of various extremophiles [9]. Our previous study on the NMR-based metabolomics investigation of this bacterial strain revealed the upregulation of glucose, ribose, and fructose residues when the bacterial isolate was grown in higher concentrations of NaCl [39]. Out of the several halophilic bacteria, and in general, extremophilic organisms, EPSs are reported to have diverse sugar compositions. Importantly, glucose, ribose, fructose, and xylose sugars are commonly found in various bacterial EPSs along with a few other monosaccharides [9]. For instance, the EPS produced by *Halomonas venusta* and *Alcaligenes faecalis* isolated from the seawater of Mauritius consists of mainly fructose and glucose sugars [52]. The EPS from *Bacillus licheniformis* T14 consists of fructose, fucose, and glucose as major monosaccharides [53]. Another thermophilic bacterium, namely, *Aeribacillus pallidus* 418, has mannose, galactose, glucose, galactosamine, glucosamine, ribose, and arabinose as the monomeric units [54]. The study of a thermophilic bacterium *Rhodothermus marinus* DSM4252T revealed that the EPS consists of glucose, arabinose, and xylose [55].

The percentage of monosaccharides is calculated based on the peak area in comparison with the standard.

### 3.7. Fourier Transform-Infrared Spectroscopy (FT-IR)

**Figure 3:**
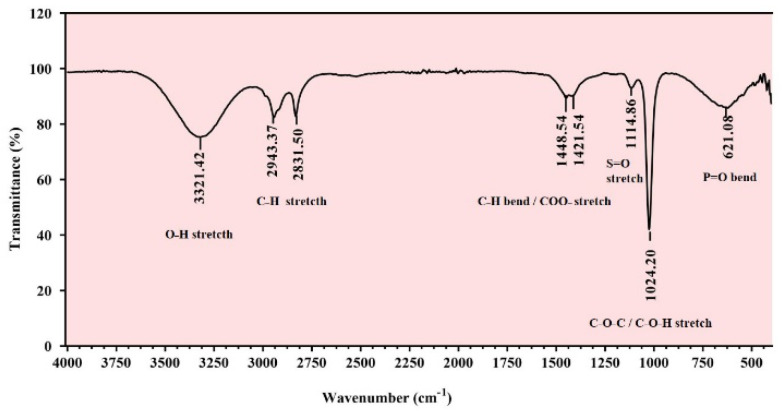
Infrared spectrum of *Virgibacillus dokdonensis* VITP14 exopolysaccharide (EPS).

FTIR spectra (Figure 3) reveal the probable functional groups present in the polymeric structure of the EPS. Previous studies of extremophiles have emphasized that the bacterial EPS consists of diverse functional groups [56]. Broad and strong absorption at 3500–3000 cm^−1^ indicates the presence of hydroxyl groups of carbohydrates [57]. Strong absorption in the region of 2943.37–2831.50 cm^−1^ is associated with a symmetric and asymmetric stretch of the C-H bond, which is a common feature in polysaccharides [58,59]. Weak peaks around 1448.54 cm^−1^ and 1421.54 cm^−1^ are attributed to bending vibrations of C-H bonds. However, there are reports that these may also be associated with stretching vibrations of COO– groups [60,61]. Additionally, the stretching of sulfate groups appears in the region 1100 to 1150 cm^−1^ [44,62]; thus, the band at 1114.86 cm^−1^ may indicate the possible presence of sulfate groups. The absorption peak around 1000–1200 cm^−1^ (C–O–H, C–O–C, and C–O) is also a characteristic feature of sugar derivatives (Figure 3). Additionally, bands from 800 to 1200 cm^−1^ are specific for polysaccharides. The region below 1000 cm^−1^ is normally assigned to bending vibrations of phosphate groups. Thus, the small peak at 621.08 cm^−1^ may correspond to the presence of the phosphate group [63]. Therefore, it is envisaged that the EPS identified in the present study has various functional groups, especially anionic groups, which correlate with the observed negative zeta potential value, and the presence of these specific functional groups influence the biological activities of the EPS [64].

### 3.8. Molecular Weight

**Figure 4:**
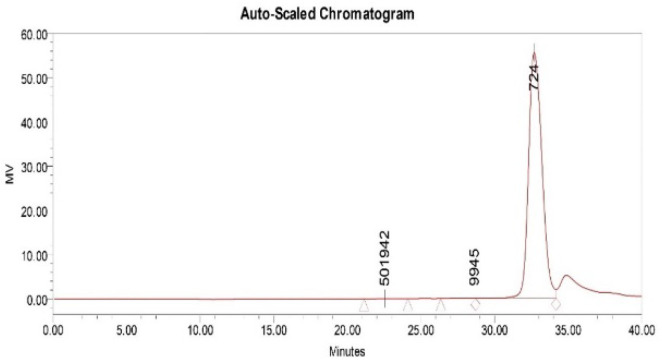
Size-exclusion chromatogram of *Virgibacillus dokdonensis* VITP14 exopolysaccharide (EPS).

The molecular weight of the EPS produced by VITP14 was determined by gel permeation chromatography, and the chromatogram is given in Figure 4. The weight-average molecular weight (*M*w) and the number-average molecular weight (*M*n) of the VIT14 EPS are 555.05 kDa and 479.70 kDa, respectively. The polydispersity index (PI) of the EPS is 1.16, which means a narrow distribution of the molecular mass (about 555 kDa) of the EPS. Moreover, the molecular weight of VITP14 EPS was found to be close to commercial anionic polymer gellan (bacterial EPS from *Sphingomonas* and *Pseudomonas* genera) whose molecular weight is 500 kDa [65]. Another anionic EPS from a marine bacterium, namely, *Alteromonas* sp. PRIM-28, has a molecular weight of 780 kDa [63]. Additionally, the previous exploration of microbial EPSs from extreme marine habitats demonstrated that the average molecular weight of EPSs is generally between 100 and 300 kDa [66]. The Mw value of 555 kDa for VIT14 EPS is within the range reported for commercial EPSs such as xanthan, alginate, and hyaluronic acid (300–50,000 kDa) [67].

### 3.9. NMR Analysis

**Figure 5:**
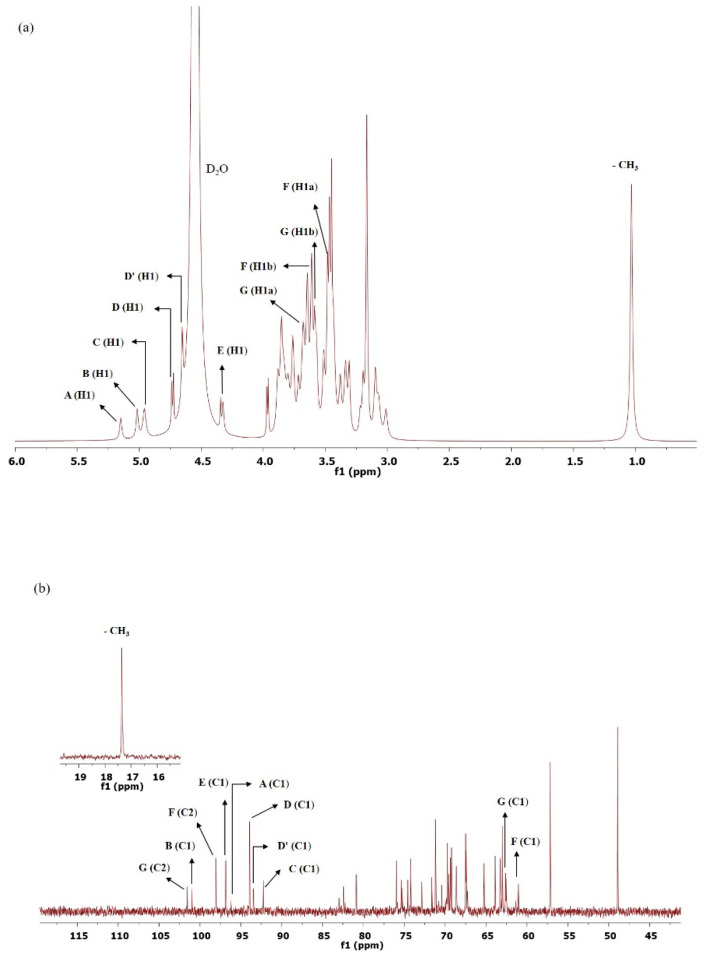

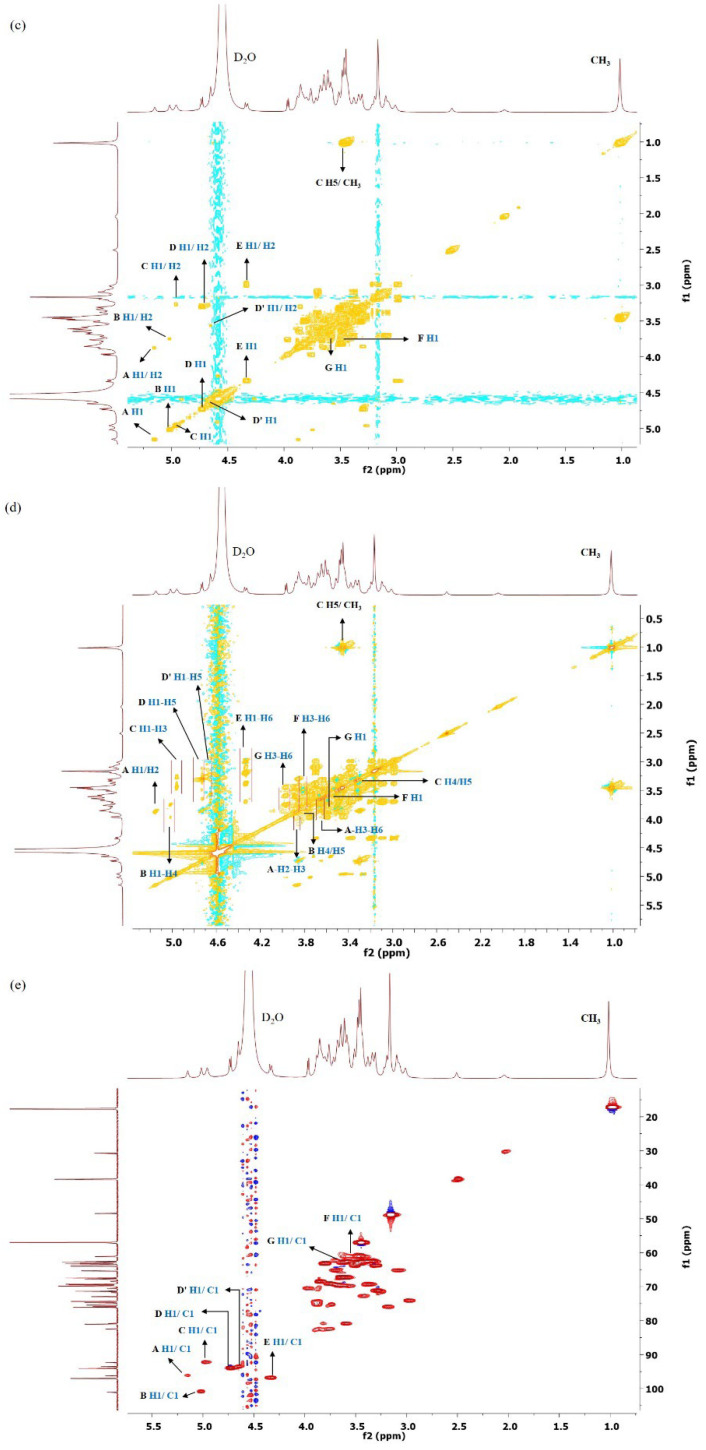

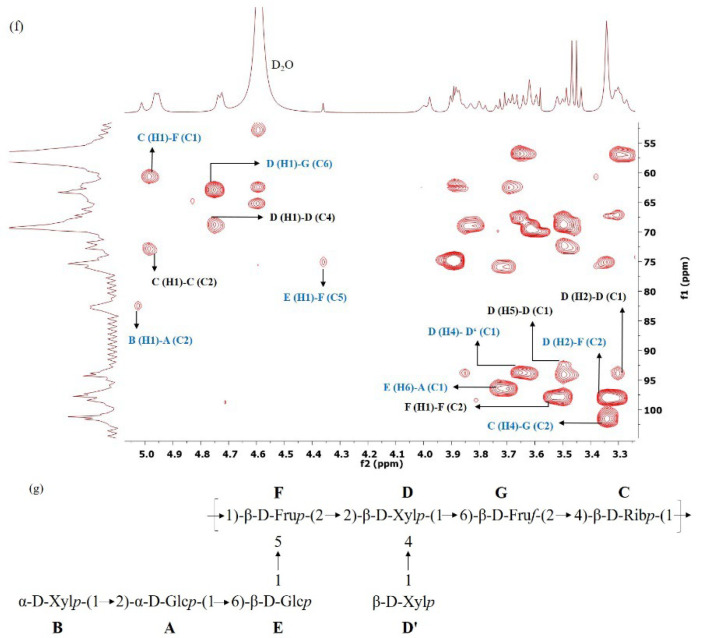
(**a**) ^1^H NMR spectrum and (**b**) ^13^C NMR spectrum; (**c**) COSY Spectrum; (**d**) TOCSY spectrum; (**e**) HSQC spectrum; (**f**) HMBC spectrum (blue—anomeric to non-anomeric linkages of different sugar residues; black—anomeric linkage of same sugar residue); (**g**) structure of EPS of *Virgibacillus dokdonensis* VITP14 exopolysaccharide (EPS).

NMR was used to determine the structural characteristics of VIT14 EPS. The number of monosaccharide units present in the EPS was determined based on the number of anomeric proton signals (designated from A to F). Thus, the signals at δ5.15, 5.02, 4.96, 4.72/4.74 (doublet), 4.66, and 4.32/4.34 ppm (doublet) in the ^1^H NMR spectrum were assigned to the anomeric protons of monosaccharides (Figure 5a). In general, chemical shifts greater than δ5.0 ppm are indicative of the α-configuration of the monosaccharides, whereas chemical shifts less than δ5.0 ppm are indicative of the β-configuration of the monomeric units [68]. The ^13^C resonances (Figure 5b) at δ92–101 ppm are attributed to anomeric carbons (C-1), whereas the signals at δ50–85 ppm are attributed to ring carbons (C-2 to C-5) of the EPS [69]. Additionally, the anomeric carbon chemical shifts assist to confirm the pyranose and furanose forms of sugar residues [70]. Further, the constituent monosaccharides were identified based on the spin system patterns observed in the COSY (Figure 5c) and TOCSY (Figure 5d) spectrum. Moreover, connectivity in the 2D-TOCSY trace was used in the preliminary identification of the relative configuration of monosaccharides [70]. Thus, based on the through-bond connectivities and the spin systems, the monosaccharide units present in VITP14 EPS are α-glucose (A), α-xylose (B), β-ribose (C), β-xylose (D and D′), β-glucose (E), and β-fructose (F and G). The ^1^H-^13^C HSQC spectrum was used to assign the carbons of the monosaccharide units (Figure 5e). Additionally, chemical shift patterns of ^1^H and ^13^C indicate that glucose and xylose exist in both α- and β-configurations as independent repeating units. The observed proton chemical shifts of monosaccharide residues of VITP14 EPS are consistent with the EPS reported in the literature [71,72,73,74,75]. The α-configuration of residues **A** and **B** and the β-configuration of residues **C**, **D**, **D′, and E** were confirmed based on the chemical shifts. These data indicate that VITP14 EPS accommodates both α- and β-linked pyranosidic residues. Further, the ^1^H, 2D-COSY, and TOCSY spectra revealed that ^1^H chemical shifts at δ3.48, 3.61 ppm correspond to H-1a, 1b protons of the β-D-fructopyranosyl (**F**-β-D-Fru*p*) residue, where their proton J-coupling constants were 40.0 Hz and 10.6 Hz, and the chemical shifts at δ3.71, 3.59 ppm correspond to H-1a, 1b protons of the β-D-fructofuranosyl (**G**-β-D-Fru*f*) residue, where their proton J-coupling constants were 25.8 Hz and 11.8 Hz, respectively [76,77]. Besides, the proton signals present at δ3.2–4.5 ppm indicate the ring protons (H-2 to H-6) of the various monosaccharides [59,78]. The signal at δ1.0 ppm in ^1^H NMR could be a putative methyl group (Figure 5a) in the EPS attached to the C5 of the ribose moiety as per the cross peaks in the COSY, TOCSY, and ^1^H-^13^C HSQC spectra.

According to the reports, the heteronuclear one-bond proton-carbon coupling constants (^1^JCH) provide information related to structure, stereochemistry, and conformation in natural products, especially in polysaccharides. For instance, (^1^JCH) coupling constants were identified for α-D-arabinopyranose sugar [79,80]. The anomeric configuration of several EPSs was identified using the (^1^JCH) coupling constants [81,82,83]. In the present study, the configuration of the anomeric position of residues A through E was confirmed by measuring the (^1^JCH) coupling constants (Table 3) obtained from the F1-coupled HSQC spectra (data not shown).

It has been reported that fructose exhibits various tautomeric forms [84]. Based on this fact, fructose in the EPS was found to be in pyranose and furanose forms. Furthermore, the C1 signals of the β-D-fructopyranosyl (F) and β-D-fructofuranosyl (G) residues were found in the region of δ60–62 ppm, whereas the anomeric C2 signals were observed at δ98 and 101 ppm in the ^13^C NMR spectrum (Figure 5b) and their cross-peaks were identified in the HMBC spectrum, respectively (Figure 5f).

The HMBC spectrum was used to determine the linkages between the monomeric residues. The HMBC spectra of the EPS displayed correlations between anomeric protons and anomeric carbons with the linkage atoms in the subsequent monosaccharide residues (Figure 5f). The monosaccharide residues were observed to have different linkage patterns (anomeric to non-anomeric) in the EPS. Inter-residue connectivities are given in Table 4. Further, the arrangement of the repeating units in VITP14 EPS (Figure 5g) was obtained from the long-range ^1^H-^13^C HMBC spectrum. Thus, the molecular structure of VITP14 EPS consists of α- and β-glycosidic configurations, with linkage patterns as shown in Figure 5f. All these different types of linkage patterns are present in other microbial polysaccharides reported in the literature, as well as in the Carbohydrate Structure Database (CSDB) [85].

### 3.10. Thermogravimetric Analysis

**Figure 6:**
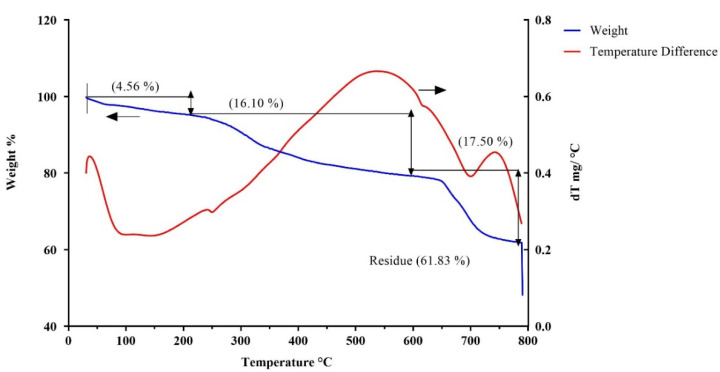
TGA/DTA—thermogram of *Virgibacillus dokdonensis* VITP14 exopolysaccharide (EPS) with three-stage decomposition. Initial temperature of 28 °C to final temperature of 800 °C.

In general, polymers undergo two- or three-stage decomposition depending on their structural complexity. TGA and DTA profiles are presented in Figure 6. EPSs, in the present study, exhibit a three-stage decomposition in the TGA analysis (Figure 6). The first phase presents a weight loss of 4.5% between 30 and 220 °C due to residual moisture loss. Above this temperature, the weight remains constant until a second degradation process occurs due to the depolymerization of the EPS with a weight loss of 16.1% observed between 220 °C and 600 °C. Between 600 °C and 800 °C, a final weight loss of 17.5% is noted. The main weight loss was observed at 251.4 °C and, therefore, VITP14 EPS is considered to be stable up to 251.4 °C. The EPS from a moderately halophilic bacterium, *Halomonas xianhensis* SUR308, displayed thermal stability up to 250 °C [86]. The gradual and slow decomposition of VITP14 EPS may be due to the presence of a thermally stable saccharide moiety and heterogeneous molecular structure. Therefore, the EPS produced by the VITP14 isolate offers great potential for exploitation in biotechnological applications requiring high thermal stability.

### 3.11. X-ray Diffraction Analysis

XRD is a powerful analytical technique for qualitative and semiquantitative analysis of the amorphous and crystalline nature of substances. The XRD of VITP14 EPS (Supplementary Figure S7) reveals sharp peaks in the range from 20 to 70 with a 2θ value. The strong and narrow peaks indicate the crystalline form of the EPS. The distinct peaks are observed at 18.90, 19.27, 23.87, 25.76, 28.32, 29.26, 32.11, 34.14, and 49.01 in the 2θ area. It has been suggested that the relative intensities of the diffraction peaks are directly proportional to the CI of the compound. The XRD pattern revealed a semicrystalline nature of the EPS (54.2%); CI_xrd_ = 0.542. The crystalline component of the material also has a greater impact on its thermal characteristics.

### 3.12. Water Solubility, Water-Holding Capacity, and Emulsifying Activity

The water solubility value of VITP14 EPS is 76.5% at 500 mg/mL, indicating that the EPS has good water solubility at a higher concentration and is better than the EPS produced by the thermophilic bacterium *Anoxybacillus gonensis* YK25, whose water solubility is 76% at 100 mg/mL [61]. Several factors such as glycosidic linkage, chain length, and structural arrangements affect the water solubility of EPSs [50]. Besides, water-holding properties are attributed to intensive hydrogen bonding and the absorptive structure of polysaccharide chains, by which a large amount of water can be retained. Based on the study, VITP14 EPS has a desirable water-holding capacity (WHC) of 266.8% when compared to the EPS of a thermophilic *Bacillus haynesii* CamB6, whose WHC was 102.9% at a similar concentration of 500 mg/mL [87]. The surface porosity, permeability, swelling abilities, and the hydroxyl groups of these EPSs are attributable to their water-holding properties. The water-holding capacity of biopolymers is preferred in synthesizing polymer-based hydrogel biomaterials [88].

The emulsifying activity of the EPS was determined against olive oil for 24 h and 48 h. The emulsifying index of the EPS at 24 h is 66.8% and is slightly reduced to 59.0% at 48 h. It has been inferred that the capacity of any emulsifier to maintain 50% of the original volume of the emulsion after 24 h could be an acceptable emulsifier. The surface-active residues in the emulsifiers modify the rheological properties of the bulk phase and reduce interfacial tension at the oil–water interface [50]. Therefore, EPS has an emulsion-stabilizing ability between water and hydrophobic compounds. Additionally, the emulsifying index of Tween 80 is 86.9% (24 h) and 79.6% (48 h). The emulsifying activity of the EPS is attributed to the functional groups and different proportions of monosaccharides in the EPS and the charges present in them. Microbial polysaccharides are considered suitable emulsifiers due to their lesser toxicity, biodegradability, and ability to stabilize emulsions in oil recovery, food stabilization, and cosmetic applications. Overall, the results suggest that VITP14 EPS has the required physicochemical properties which are suitable for biotechnological applications.

### 3.13. Hemocompatibility and Erythrocyte Membrane Stabilization Activity of EPS

**Figure 7:**
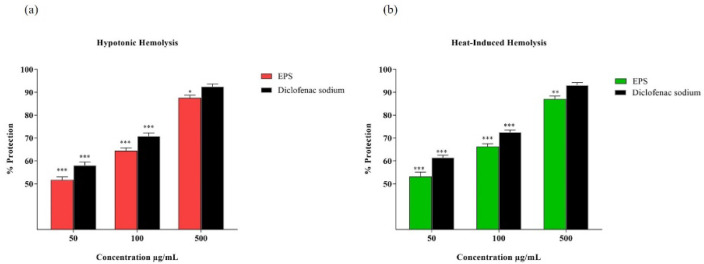
Membrane stabilization activity of *Virgibacillus dokdonensis* VITP14 exopolysaccharide (EPS): (**a**) hypotonicity; (**b**) heat-induced erythrocyte lysis. Values are means and error bars indicate standard deviations (*n* = 3). The results *p* < 0.05 (*), *p* < 0.01 (**), *p* < 0.001 (***) were considered as significant in comparison to control.

Hemocompatibility of biomaterials is one of the most essential criteria for the fabrication of biomaterials [89]. Importantly, the erythrocyte hemolysis test is considered a reliable method of analyzing blood compatibility and the preliminary toxicity of materials. Thus, non-hemolytic activity and the inhibition of hemolysis (membrane stabilization) were measured to validate the hemocompatibility of the EPS. The effect of the EPS on erythrocytes was evaluated by hemolysis assay. Results indicate that VITP14 EPS does not exhibit any harmful effects on erythrocytes when used alone. Based on the quantification, the hemolysis rate of the EPS at different concentrations was less than 4% (data not shown). It is suggested that the rate of hemolysis which is less than 5% is permissible for biomedical materials as per the authoritative assessment criteria (ASTM F756-17) [90]. In the assessment of membrane-stabilizing ability, it is observed that the EPS confers 64.38% (100 μg/mL EPS) and 87.48% (500 μg/mL EPS) protection against hypotonic-induced lysis of the erythrocyte membrane (Figure 7a). This observation is comparable to that of diclofenac sodium (70.78 and 92.43%, at 100 and 500 μg/mL, respectively). Both the EPS and diclofenac sodium exhibit a concentration-dependent inhibition against hypotonic lysis. In the case of heat-induced hemolysis, the EPS resists hemolysis as the standard drug used. The hemolytic-inhibiting potential at 100 and 500 μg/mL is 66.22% and 87.06%, respectively (Figure 7b). Comparatively, diclofenac sodium at 100 and 500 μg/mL has a maximum protection of about 72.44% and 93.05%, respectively.

Human red blood cell (RBC) membranes are similar to lysosomal membranes. Membrane stabilization is a process that helps in maintaining the integrity of biological membranes such as lysosomal membranes against osmotic stress and heat-induced lysis. This hypotonic solution can cause the excessive accumulation of fluid within cells that ruptures the membrane and leads to osmotic loss. Therefore, membrane stabilization can prevent the leakage of fluids and serum protein into the tissues during enhanced permeability, which is caused by inflammatory mediators [91]. The possible mechanism for the stabilization property is attributed to the binding of the EPS to membrane proteins, which possibly alter the surface volume ratio of the erythrocyte membrane, thus modifying the surface charge and calcium flow in the membranes and preventing the aggregating agents [92]. The EPS also protects erythrocyte hemolysis similar to that of other natural compounds. For instance, the EPS extracted from the psychotropic bacterium *Pseudomonas* sp. BGI-2 inhibits hemolysis induced by Triton-X and SDS (sodium dodecyl sulfate) [93]. Similarly, the EPS produced by the endophytic fungus *Fusarium solani* SD5 inhibits hyposaline-induced hemolysis of erythrocytes (55.05%) at 1000 µg/mL [94]. Thus, the results indicate that VITP14 EPS is not toxic to RBCs, has the potential to stabilize the RBC membrane, and can be considered an effective membrane stabilizer at higher concentrations. The results suggest that EPSs possess good biocompatibility.

### 3.14. Anticoagulant Activity of EPS

Many biomaterials induce thrombosis or foreign body reactions. In most cases, blood coagulation occurs when blood cells contact the surface of the biomaterial. [95]. Therefore, developing antithrombotic biomaterials has become an emerging trend in industries to improve their hemocompatibility [96]. Hence, the evaluation of anticoagulation potential or property is an important step during the selection of biomaterials, especially blood-contacting biomaterials and implants. In the present study, the effects of the EPS on APTT and PT are evaluated (Supplementary Table S3). VITP14 EPS significantly prolongs the APTT and PT, indicating anticoagulant ability. APTT was extended over 40 s at 100 µg/mL and 120 s at 500 µg/mL of the EPS. The anticoagulant effect of the EPS at 100 and 500 µg/mL concentrations extended the PT beyond 20 s and 60 s, respectively. Comparatively, heparin (100 µg/mL) prolongs APTT > 300 s and PT > 100 s (data not shown). Clinically, APTT and PT are often used to evaluate the coagulation function of the body. As an intrinsic coagulation pathway, APTT measures the function of coagulation factors VIII, IX, and XI, whereas PT, an extrinsic coagulation pathway, measures the function of coagulation factors I, V, VII, and X. According to the literature, the anticoagulant activity of the EPS has an impact on both extrinsic and intrinsic pathways of blood coagulation [97]. For example, the EPS from *Lactococcus lactis* F-mou has similar anticlotting activity [98]. Normally, the structural composition, configuration, molecular weight, branching structure, and sulfate groups influence the anticoagulant activities of the EPS [99]. These in vitro anticoagulant effects of VITP14 EPS are most likely to have occurred due to the electrostatic interactions and their binding ability to coagulation factors of plasma. Therefore, VITP14 EPS can be used for the formulation of biomaterials which require hemocompatibility.

**In vitro cytocompatibility**

### 3.15. Cell Viability

**Figure 8:**
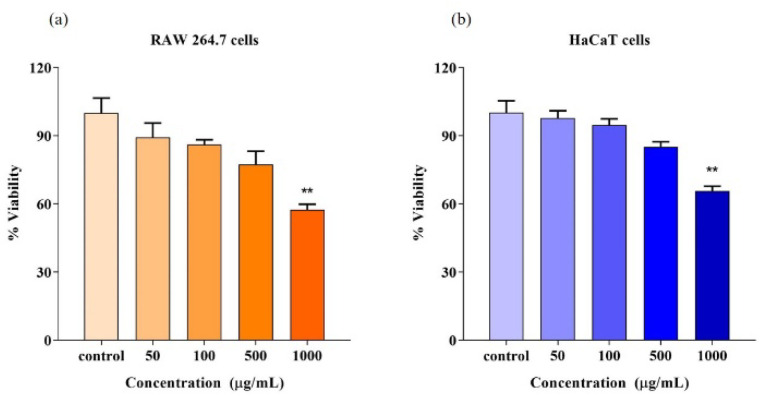
Effect of *Virgibacillus dokdonensis* VITP14 exopolysaccharide (EPS) on cell viability: (**a**) RAW264.7 cells; (**b**) HaCaT cells. Values are means and error bars indicate standard deviations (*n* = 3). The results *p* < 0.05 (*), *p* < 0.01 (**) were considered as significant in comparison to control.

Evaluation of cytotoxicity is a primary and mandatory criterion for evaluating biocompatibility. In such an evaluation, cultured cells are allowed to interact with the test material. Subsequently, the status of the cell is observed for metabolism and growth. Additionally, this is a reliable method in the selection of biomaterials for clinical applications [11]. The MTT method is normally used for analyzing the cytocompatibility of test materials. In the present study, RAW264.7 murine macrophages and human keratinocyte HaCaT cells were treated with various concentrations of the EPS to evaluate cytotoxicity. RAW264.7 macrophages are commonly used for the study of cellular responses to microbes and their products. As shown in Figure 8a, the viability of the control was set as 100%. Cells exposed to the EPS at the concentration of 50–500 µg/mL have shown a cell viability above 80%. The results suggest that the EPS does not exhibit cytotoxicity on RAW264.7 cells up to 500 μg/mL. As can be observed from Figure 8b, the viability of HaCaT cells treated with the EPS is greater than 85% at concentrations ranging from 50 to 500 μg/mL, suggesting that VITP14 EPS is not cytotoxic to HaCaT cells up to 500 μg/mL. The maximum non-cytotoxic concentration of the EPS obtained from *Lactobacillus pentosus* LZ-R-17 is 400 µg/mL [100], whereas VITP14 EPS has shown good cell viability even at 500 µg/mL. This signifies that the EPS is biocompatible and can be used in biomaterial/drug applications.

## 4. Conclusions

Halophilic bacteria are accepted as novel sources of EPSs for diverse biological activities. To our knowledge, there are no studies on EPS production, structural composition, and biological properties of the bacterial strain reported in the present study. Nevertheless, these types of bacterial species have been utilized for the production of various enzymes and other metabolites. In this study, the EPS was produced by *Virgibacillus dokdonensis* VITP14 and extracted using ethanol. The maximum production of the EPS (17.3 g/L) was observed at 96 h of fermentation. Further, the EPS was purified using ion exchange and gel permeation chromatographic methods. The microstructural analysis based on SEM and AFM revealed the fibrous, porous structure and surface roughness of the EPS. Elemental composition showed the presence of carbon, oxygen, sodium, magnesium, phosphate, and sulfate and confirmed the organic nature of the EPS. The findings indicated that the EPS is anionic (zeta potential value of −10 mV) and is composed of glucose, ribose, fructose, and xylose. The FTIR spectrum showed the presence of various functional groups (O-H, C-H, C-O-H, C-O, S=O, and P=O) in the EPS. The EPS has an average molecular weight of 555 kDa. The NMR results confirmed the presence of the sugar residues, the existence of two α- and six β-linkage types, and the branched structure of the EPS. Results showed that the EPS has a high thermal stability (251.4 °C) and is semicrystalline. Moreover, the EPS was demonstrated to have good water solubility, water-holding capacity, and emulsifying properties. The EPS is non-toxic and exhibits significant RBC membrane-stabilizing potential, anticoagulant activity (APTT and PT), and cytocompatibility on RAW264.7 macrophages and keratinocyte HaCaT cells. In summary, this study demonstrates that VITP14 EPS is biocompatible and addresses the rising demand for natural polysaccharides in various biotechnological applications, especially in the formulation of biomaterials.

## Figures and Tables

**Table 1:** Exopolysaccharide (EPS) production on Zobell marine broth by VITP14.

S. No	Time (h)	Dry Cell Weight (g L^−1^)	EPS (g L^−1^)
1	24	8.253 ± 0.533	9.327 ± 0.621
2	48	12.307 ± 0.603	13.333 ± 0.474
3	72	13.927 ± 0.783	15.680 ± 0.675
4	96	13.260 ± 0.623	17.333 ± 0.565

**Table 2:** Monosaccharide composition of exopolysaccharides (EPSs).

S. No	Monosaccharides	VITP14 EPS (%)
1	Glucose	25.8
2	Ribose	18.6
3	Fructose	31.5
4	Xylose	24.0

**Table 3:** ^1^H and ^13^C NMR chemical shifts (δ, ppm) of EPS.

Residue	H1/C1	H2/C2	H3/C3	H4/C4	H5/C5	H6/C6	CH_3_
**A**) → 2)-α-D-Glcp-(1 →	5.15 96.07 (180 Hz)	3.87 82.45	3.80 82.60	3.73 75.35	3.82 80.83	3.68 70.02	- -
**B**) α-D-Xylp-(1 →	5.02 100.86 (183 Hz)	3.75 82.41	3.96 71.56	3.83 74.08	3.57 69.93	- -	- -
**C**) → 2,4)-β-D-Ribp-(1 →	4.96 92.22 (165 Hz)	3.28 72.20	3.47 71.80	3.34 69.65	3.44 65.03	- -	1.03 17.19
**D**) → 2,4,5)-β-D-Xylp-(1 →	4.72/4.74 93.90 (163 Hz)	3.30 72.31	3.85 70.10	3.65 70.64	3.49 67.65	- -	- -
**D′**) β-D-Xylp-(1 →	4.66 93.46 (161 Hz)	3.56 70.31	3.72 70.11	3.66 69.81	3.62 67.48	- -	- -
**E**) → 6)-β-D-Glcp-(1 →	4.32/4.34 96.78 (159 Hz)	3.00 74.13	3.11 76.01	3.20 75.92	3.37 69.56	3.70 67.92	- -
**F**) → 1)-β-D-Frup-(2 →	3.48/3.61 61.02	- 98.04	3.79 68.52	3.85 69.20	3.88 75.15	3.51/3.58 61.50	- -
**G**) → 6)-β-D-Fruf-(2 →	3.71/3.59 62.40	- 101.61	3.97 70.59	3.86 69.45	3.76 81.03	3.77/3.64 63.05	- -

CH_3_—methyl group; measured (^1^JCH) coupling constants are given in parentheses.

**Table 4:** Inter-residue correlations from the anomeric atoms observed in ^1^H and ^13^C HMBC spectrum of EPS.

**Residues**	**HMBC**		**Chemical Shifts**
From	To	Linkage	(δ, ppm)
**A** (H-1) **A** (C1)	**E** (C-6) **E** (H-6)	**A** (1 → 6) **E** α-D-Glcp-(1 → 6)-β-D-Glcp	**AC1** (96.17)–**EH6** (3.70)
**B** (H-1) **B** (C1)	**A** (C-2) **A** (H-2)	**B** (1 → 2) **A** α-D-Xylp-(1 → 2)-α-D-Glcp	**BC1** (5.02)–**AH2** (82.45)
**C** (H-1) **C** (C1)	**F** (C-1) **F** (H1)	**C** (1 → 1) **F** β-D-Ribp-(1 → 1)-β-D-Frup	**CH1** (4.96)–**FC1** (61.00)
**D** (H1) **D** (C1)	**G** (C-6) **G** (H-6)	**D** (1 → 6) **G** β-D-Xylp-(1 → 6)-β-D-Fruf	**DH1** (4.72)–**GC6** (63.10)
**D′** (H1) **D′** (C1)	**D** (C-4) **D** (H-4)	**D′** (1 → 4) **D** β-D-Xylp-(1 → 4)-β-D-Xylp	**D′C1** (93.60)–**DH4** (3.65)
**E** (H1) **E** (C1)	**F** (C-5) **F** (H-5)	**E** (1 → 5) **F** β-D-Glcp-(1 → 5)-β-D-Frup	**EH1** (4.34)–**FC5** (75.13)
**F** (nd) **F** (C2)	**D** (C-2) **D** (H-2)	**F** (2 → 2) **D** β-D-Frup-(2 → 2)-β-D-Xylp	**FC2** (98.01)–**DH2** (3.30)
**G** (nd) **G** (C2)	**C** (C-4) **C** (H-4)	**G** (2 → 4) **C** β-D-Fruf-(2 → 4)-β-D-Ribp	**GC2** (101.61)–**CH4** (3.34)

Nd—not detected.

## Data Availability

Not applicable.

## Supplementary Materials

The following supporting information can be downloaded at: https://www.mdpi.com/article/10.3390/polym14193986/s1, Table S1: Total carbohydrate and proteins in EPS; Table S2: EPS production of different strains from different locations that are compared to the strains of the present study; Table S3: Anticoagulant activity of EPS based on APTT and PT; Figure S1: (**a**) Mucoid colonies of *Virgibacillus dokdonensis* VITP14 in Zobell marine agar; (**b**) Morphology of *Virgibacillus dokdonensis* VITP14 after Gram staining procedure under light microscope 100×; Figure S2: (**a**) EPS production by different carbon sources; (**b**) EPS production under different salt conditions; Figure S3: (**a**) Elution curve of EPS fractions in DEAE–cellulose 52 anion-exchange column purified by different concentrations of NaCl (0.1–1.1 M); (**b**) Elution curve of EPS on Sephacryl S-300 gel chromatography column with distilled water; Figure S4: AFM analysis of EPS (3D view) 800 nm scale; Figure S5: Zeta potential of EPS; Figure S6: (**a**) The HPLC results of Virgibacillus dokdonensis VITP14 exopolysaccharide (EPS) (glucose—5.143 min, ribose—5.593 min, fructose—13.236 min, xylose—13.616 min); Figure S6: (**b**) The HPLC chromatographic peaks of sugar standards: peak 1—arabinose, peak 2—glucose (5.502 min), peak 3—galactose (5.605 min), peak 4—mannose (5.807 min), peak 5—ribose (5.884 min), peak 6—xylose (13.916 min), peak 7—fructose (13.104 min); Figure S7: XRD profile of EPS.
