# Supplementary material for: Molecular Characterization and Biocompatibility of Exopolysaccharide Produced by Moderately Halophilic Bacterium Virgibacillus dokdonensis from the Saltern of Kumta Coast
Source: Polymers (Basel). 2022 Sep 23;14(19):3986. doi: 10.3390/polym14193986 (PMC9570845; doi:10.3390/polym14193986)
Supplement: Supplementary file 1 [file polymers-14-03986-s001.zip › polymers-1900611-supplementary.pdf]

SUPPLEMENTARY MATERIALS

Molecular Characterization and Biocompatibility of Exopolysaccharide Produced by Moderately Halophilic bacterium *Virgibacillus dokdonensis* from the Saltern of Kumta Coast

Affiliation

***Gurunathan Jayaraman-** Department of Biotechnology, School of Biosciences and Technology, Vellore Institute of Technology, Vellore, Tamil Nadu, India 632014

# Monic Andrew- Department of Biotechnology, School of Biosciences and Technology, Vellore Institute of Technology, Vellore, Tamil Nadu, India 632014

***Corresponding author:** Dr G.Jayaraman, E-mail: gjayaraman@vit.ac.in. Tel: + 91416 2202011. Fax: + 91 416 224 3092

List of Supplementary Tables

Supplementary Table S1. Total carbohydrate and proteins in EPS;

Supplementary Table S2. EPS production of different strains from different locations that are compared to the strains of present study

Supplementary Table S3. Anti-coagulant activity of EPS based on APTT and PT

List of Supplementary Figures

Supplementary Figure S1. **(a)** Mucoid colonies of *Virgibacillus dokdonensis*-VITP14 in Zobell marine agar; **(b)** Morphology of *Virgibacillus dokdonensis*-VITP14 after Gram staining procedure under light microscope 100×.

Supplementary Figure S2. (a) EPS production by different carbon sources. **(b)** EPS production under different salt conditions

Supplementary Figure S3. (a) Elution curve of EPS fractions in DEAE–cellulose 52 anion-exchange column purified by different concentrations of NaCl (0.1-1.1M); **(b)** Elution curve of EPS on Sephacryl S-300 gel chromatography column with distilled water.

Supplementary Figure S4. AFM analysis of EPS (3D view) 800nm scale

Supplementary Figure S5. Zeta potential of EPS

Supplementary Figure S6. (a) The HPLC results of *Virgibacillus dokdonensis* VITP14 Exopolysaccharide (EPS) (glucose – 5.143 min; ribose – 5.593 min; fructose – 13.236 min; xylose – 13.616 min; **(b)** The HPLC chromatographic peaks of sugar standards: peak 1- arabinose; peak 2 – glucose (5.502 min); peak 3 – galactose

(5.605 min); peak 4 – mannose (5.807 min); peak 5 – ribose (5.884 min); peak 6 – xylose (13.916 min); peak 7 – fructose (13.104 min).

Supplementary Figure S7. XRD profile of EPS

Total: Tables (3)

Table S1. Total carbohydrate and proteins in Exopolysaccharides (EPS)

**Strain**	**Final concentration CHO (% w/w)**	**Protein (% w/w)**
Virgibacillus sp VITP14	96.75.10 ± 0.76	0.47 ± 0.10

Table S2. EPS production of different strains from different locations that are compared to the strains of present study

**S.No**	**Microorganism**	**Source**	**Growth Medium**	**EPS Yield (g/L)**	**Reference**
1	*Pantoea* sp. *BM39*	Tyrrhenian Sea	EMG medium	21.3	(Silvi et al 2013)
2	*Halobacillus* sp *EG1HP4QL*	Lake Qarun, Egypt	S-G medium, Sucrose	5.9	(Ibrahim et al 2020)
3	*Halomonas hydrothermalis* MB45	South Indian Ocean	Zobell marine broth	5.2	(Banerjee et al 2020)
4	*Rhodobacter johrii.*	Cabo-De-Rama beach Goa, India	R2A medium with Glucose	6.2	(Sran Kulwinder Singh et al 2019)
5	*E. cloacae* VVD-MBB8	Gulf of Mannar, Tamil Nadu, India.	Modified Minimal media	18.3	(Karuppiah et al 2021)
6	***Virgibacillus Dockdonensis VITP14***	Saltern Kumta coast, Arabian Sea, India	Zobell Marine broth	17.3	**Present study**
			Zobell Marine broth + glucose	23.2	
7	*Salibacterium halochares STm*	Cuatro Ciénegas Basin, Coahuila Mexico	yeast extract and NaCl	17.3	(Lopez et al 2021)
8	*Chromohalobacter salexigens*	Lake Qarun, Egypt	Sucrose	15.1	(Ibrahim et al 2022)

Table S3. Anti-coagulant activity of EPS based on APTT and PT

S.No	EPS concentration µg/mL	APTT (seconds)	PT (seconds)
1	Control	32.4 ± 1.8	13.8 ±1.9
2	50	28.6 ± 1.4^{ns}	13.6 ± 0.8^{ns}
3	100	43.2 ± 1.9^{b}	24.7 ± 1.2^{a}
4	500	123.3 ± 1.9^{a}	61.4 ± 1.5^{a}

Note: ns-non significant; a-(*p*<0.0001) b-(*p*<0.001) were statistically significant in comparison to control

Figures (7)

Figure S1:

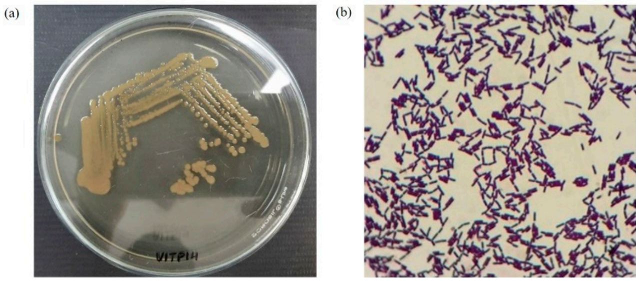

Figure S1. (a) Mucoid colonies of *Virgibacillus dokdonensis* VITP14; **(b)** Morphology of *Virgibacillus dokdonensis*-VITP14 (100X Magnification under Light Microscope)

Figure S2

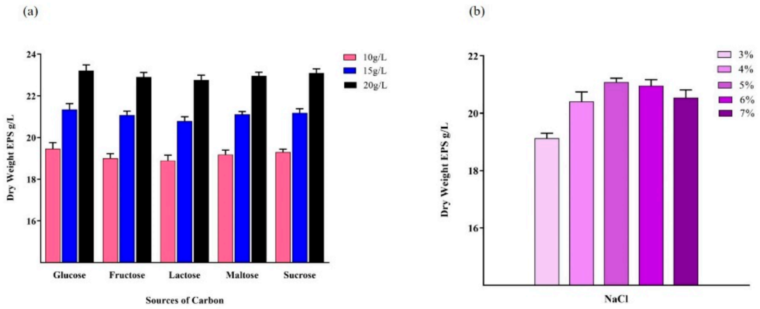

Figure S2. (a) EPS production by different carbon sources; **(b)** EPS production under different salt conditions

Figure S3

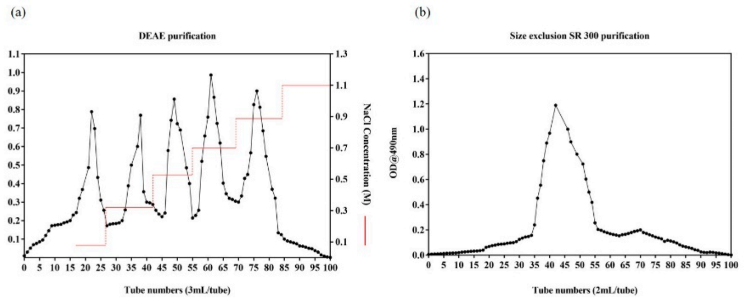

Figure S3. (a) Elution curve of EPS fractions in DEAE–cellulose 52 anion-exchange column purified by different concentrations of NaCl (0.1-1.1M); **(b)** Elution curve of EPS on Sephacryl S-300 gel chromatography column with distilled water.

Figure S4

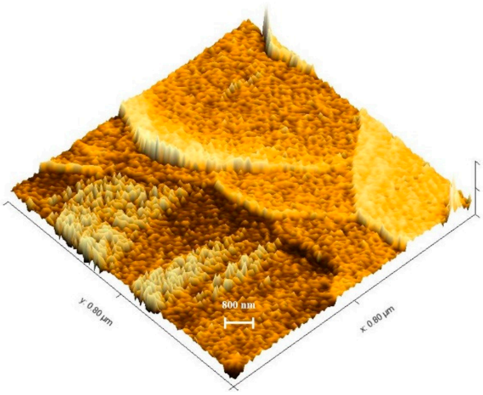

Figure S4. AFM analysis of EPS (3D view) 800nm scale

Figure S5

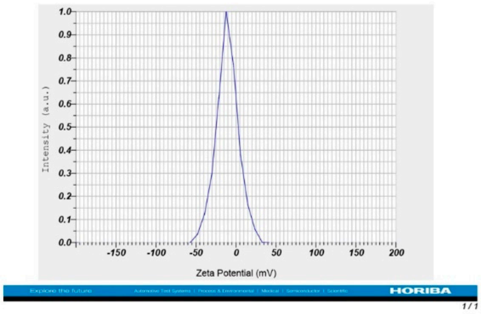

Figure S5. Zeta potential of EPS

Figure S6:

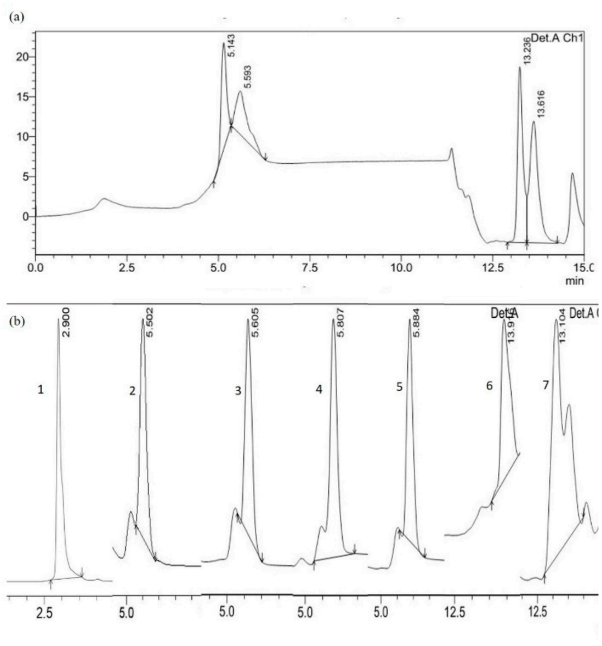

Figure S6. (a) The HPLC results of *Virgibacillus dokdonensis* VITP14 Exopolysaccharide (EPS) (glucose – 5.143 min; ribose – 5.593 min; fructose – 13.236 min; xylose – 13.616 min; **(b)** The HPLC chromatographic peaks of sugar standards: peak 1- arabinose; peak 2 – glucose (5.502 min); peak 3 – galactose (5.605 min); peak 4 – mannose (5.807 min); peak 5 – ribose (5.884 min); peak 6 – xylose (13.916 min); peak 7 – fructose (13.104 min).

Figure S7:

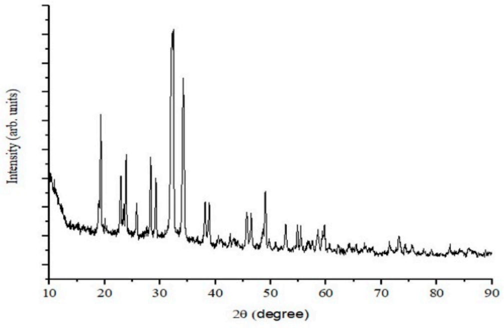

Figure S7. XRD profile of EPS
